# A bi-objective robust optimization model for location-transportation under uncertainty with psychological costs

**DOI:** 10.1371/journal.pone.0340058

**Published:** 2026-01-12

**Authors:** Tingting Zhang, Yanqiu Liu, Zhongqi Peng

**Affiliations:** School of Management, Shenyang University of Technology, Shenyang, China; Mississippi State University, UNITED STATES OF AMERICA

## Abstract

After an earthquake, it is often difficult to obtain accurate information on the number of casualties in the disaster area, which brings significant uncertainty to emergency rescue logistics. In addition to physical injuries, victims often suffer from severe psychological trauma, further hindering the effectiveness of rescue efforts. As such, incorporating the psychological condition of casualties is essential to developing an effective rescue strategy under uncertainty in casualty numbers. Building on this, this paper proposes a bi-objective robust optimization model to determine the optimal locations of medical facilities and the transportation plans for the casualties within a three-tier rescue chain comprising disaster areas, temporary hospitals, and comprehensive hospitals. The Injury Severity Score (ISS) is employed to classify casualties into three categories and to give the dynamic evolution of the deterioration rate of casualties over time. The model also considers factors such as limited medical resources, casualty classification, uncertainty in casualty numbers, and psychological conditions. The objective is to minimize the total ISS and psychological cost. We use the robust optimization method to derive the robust counterpart model of the proposed stochastic model. The bi-objective model is solved using the ε−constraint method. Extensive computational experiments and sensitivity analyses based on the Yushu earthquake were conducted, and the main findings are as follows. The greater the uncertainty in casualty numbers, the more significant its impact on the total ISS. While greater attention to the psychological condition of casualties can improve humanitarian care, it may reduce rescue efficiency, and decision-makers need to make a trade-off based on actual preferences. The treatment needs of serious casualties deserve greater attention than those of moderate or mild ones. Moreover, expanding the capacity of temporary hospitals is more effective in improving rescue efficiency than comprehensive hospitals. Finally, the robust optimization model performs better than the deterministic model when expanding the problem size.

## 1 Introduction

Earthquakes are among the most destructive natural disasters. They occur frequently, pose a serious threat to human life, and impact economic and social development. Throughout history, many major earthquakes have caused severe damage to the affected regions. For example, on February 6, 2023, a magnitude 7.8 earthquake occurred in Gaziantep Province, Turkey, killing at least thousands of people and injuring more than 10,000 people. On May 5, 2023, an earthquake of magnitude 6.5 occurred in the Noto Peninsula, Ishikawa Prefecture, Japan, causing hundreds of casualties and many buildings to collapse or be damaged. On January 22, 2024, a magnitude 7.1 earthquake occurred in Wushi County, Aksu Prefecture, Xinjiang, China, with a focal depth of 22 kilometers, killing three people and injuring five people. Due to their suddenness and destructiveness, disasters often result in a large number of casualties, creating an urgent need for casualty transfer and emergency medical treatment. In many cases, the number of casualties rises sharply in the aftermath, far exceeding the capacity of local medical services and resulting in a severe shortage of rescue resources. To address this challenge, it is crucial to rapidly formulate an effective rescue plan, optimize casualty transportation, and allocate medical resources efficiently to mitigate the impacts of disasters and improve rescue outcomes.

The key to post-disaster emergency medical rescue is ensuring that casualties receive timely treatment, thereby effectively reducing the mortality rate caused by the disaster. The “on-site treatment” model is crucial for improving rescue efficiency. By setting up temporary hospitals at health centers or clinics near the disaster area, initial treatment can be provided to casualties, reducing the risk of secondary injuries from long-distance transportation. Afterward, casualties are transferred to comprehensive hospitals for further emergency medical care. However, the destruction of communication facilities, blocked roads, and the complex disaster environment, compounded by delayed information transmission, make it extremely difficult to accurately assess the number of casualties in the disaster area. In this context, decision-makers need to determine the optimal locations for temporary and comprehensive hospitals, as well as the transfer and allocation of casualties, considering the uncertainty of the number of casualties. Additionally, to improve survival rates and rescue efficiency, decision-makers must classify casualties based on the severity of their injuries and ensure effective transport and treatment with limited resources. Commonly used injury severity scoring methods include Trauma Index Score (TIS), Trauma Score (TS), Revised Trauma Score (RTS), CRAMS Scale, and Injury Severity Score (ISS) [1–5]. The ISS method is widely used in trauma assessment because of its high predictive accuracy, broad applicability, and ease of operation. It can comprehensively assess the injuries of various parts and systems. Therefore, similar to the work of [6], this paper adopts the ISS method and divides the casualties into mild, moderate, and serious categories. Meanwhile, the deterioration of casualties is evaluated based on the changes in ISS during casualty transportation.

Earthquake disasters not only cause serious physical injuries but also lead to severe psychological trauma. Survivors often suffer from various mental health issues, including post-traumatic stress disorder (PTSD), depression, anxiety, stress-related illnesses, substance abuse, and even suicidal tendencies, all of which may reduce their willingness to engage in self-rescue or accept external aid [7,8]. Therefore, psychological impacts must be incorporated into the design of rescue plans [9,10]. Despite its importance, the psychological state of casualties is often overlooked in emergency logistics research.

To this end, this study aims to answer the following questions:

(1) Under uncertainty in the number of casualties across different severity levels (mild, moderate, and serious), how can decision-makers jointly optimize the location of temporary and comprehensive hospitals together with the allocation of casualties?

(2) Under resource constraints in emergency response, how should treatment priorities be assigned across casualties with different severities, and which type of facility—temporary hospitals or comprehensive hospitals—is more critical to overall rescue effectiveness?

(3) When humanitarian concerns are taken into account, how can decision-makers make the trade-off between treatment efficiency and psychological cost?

To address the above questions, this paper develops a robust bi-objective optimization model that incorporates emergency facility location, resource allocation, and casualty transportation planning under the uncertainty of casualty numbers. The model aims to minimize the total weighted ISS and psychological cost of casualties in post-earthquake emergency logistics rescue. The robust optimization method proposed by [11] is adopted to address the uncertainty in the number of casualties in the model. Currently, many scholars employ stochastic programming to address uncertainty in emergency logistics. However, stochastic programming methods require prior knowledge of the probability distribution of uncertain parameters, which is often impractical. If the probability distribution of uncertain parameters is assumed, discrepancies from the actual distribution may lead to decisions that do not align with the real situation. In contrast, the robust optimization method treats uncertainty as interval data fluctuating around the nominal value without relying on probability distributions, allowing for flexible control over the degree of uncertainty. Furthermore, a linearly equivalent and tractable optimization model is obtained by deriving the robust counterpart of the proposed stochastic model.

The contributions of this paper are as follows: (1) A bi-objective robust optimization model is developed to jointly optimize emergency facility location and casualty transportation under uncertainty in casualty numbers. The objectives are to minimize the total ISS and the psychological cost of all casualties. Moreover, we analyze the uncertainty in the number of casualties and the impact of the capacities of comprehensive hospitals and temporary hospitals on rescue efficiency. The results provide more informed decision support for managers. (2) The ISS is introduced to describe the casualties’ injuries, and changes in their deterioration rate over time are considered. (3) Considering that existing emergency rescue studies have largely overlooked the psychological state of casualties, this paper integrates psychological cost with the ISS into a bi-objective robust optimization model to reveal the interaction between psychological factors and rescue efficiency, providing decision-makers with new perspectives and practical insights for balancing humanitarian care and operational effectiveness. (4) The robustness of the solutions generated by the robust and deterministic models is evaluated as the problem size increases.

This paper is organized as follows. Sect 2 reviews the relevant literature. Sect 3 presents the problem definition and mathematical formulation. Sect 4 reviews the applied robust optimization methods and ε−constraint methods. Sect 5 demonstrates our model through a case study of the Lushan earthquake and provides computational results. Finally, Sect 6 summarizes our findings and discusses future research directions.

## 2 Literature review

We review the relevant literature from four aspects. Sect 2.1 focuses on post-disaster logistics rescue, while Sect 2.2 examines literature on casualty transportation. In Sect 2.3, we explore studies on post-disaster rescue that incorporate the psychological factors of casualties. Finally, Sect 2.4 discusses research on post-disaster rescue problems based on robust optimization.

### 2.1 Disaster relief logistics problems

Disaster logistics and rescue involve the location of medical facilities and casualty transportation planning. Many scholars have conducted research on these issues [12–17]. Existing studies on medical facility location have predominantly focused on objectives such as minimizing transportation distance or time, or maximizing the number of casualties that can be rescued. For example, Gu et al. [18] established a facility site selection model aimed at maximizing the number of patients. Moeini et al. [19] proposed a bi-objective stochastic optimization model that simultaneously minimizes the total construction cost and the total transportation time. Hamzani et al. [20] developed an optimization model for a large-scale disaster relief location–routing problem, aiming to minimize both waiting time and total cost. For casualty transportation problems, most studies have focused on minimizing total transportation time or maximizing the number of survivors. For example, Daglayan and Karakaya [21] developed an optimized emergency vehicle scheduling model with the objectives of minimizing the number of ambulance trips and reducing the time required to transport casualties to hospitals. Mills et al. [22] considered the survival probabilities and service times of different types of casualties and developed a casualty transportation model aimed at maximizing the expected number of survivors. Gharib et al. [23] developed a casualty transportation model with the objectives of minimizing casualty waiting time and total transportation cost.

Facility location and casualty transportation have an inseparable relationship, and integrating these two issues can improve the efficiency of disaster relief logistics systems. For example, Salman and Gül [24] established a multi-period integrated facility location and casualty distribution optimization model that minimizes a weighted sum of total transportation distance, waiting time, and facility construction cost. Pouraliakbarimamaghani et al. [25] proposed an optimization model for the medical facility location and casualty allocation problem in mass casualty incidents to improve the efficiency of medical resource utilization and alleviate the pressure of hospital overload. Aghsami et al. [26] proposed a mixed-integer nonlinear programming model for facility location and personnel allocation in humanitarian logistics, aiming to minimize total pre- and post-disaster costs. However, the above studies do not consider the dynamic deterioration of casualty conditions as transportation times increase. In real disaster rescue operations, the physiological state of casualties is highly time-sensitive; delays in treatment not only accelerate deterioration but may also sharply increase the probability of death. Therefore, we incorporate the time-varying deterioration rate of casualties, thereby enhancing the practical relevance of the model and improving the effectiveness of the resulting rescue plans.

### 2.2 Casualty transportation problem on disaster relief logistics

Following large-scale natural disasters, the number of casualties often rises sharply while rescue resources in the affected areas remain severely constrained. Consequently, the timely transportation of casualties from disaster zones to emergency medical facilities becomes a critical component of effective emergency response. Currently, some scholars have conducted in-depth discussions on this issue [27–30]. For example, Dean and Nair [28] considered factors such as vehicle rescue capacity and resource constraints at medical facilities, and modeled the transportation of victims to hospitals to maximize the expected number of survivors. Talarico et al. [29] developed an ambulance route optimization model that minimizes the weighted waiting time for casualties. Caunhye et al. [30] investigated casualty transportation in the context of catastrophic radiological events, aiming to minimize transportation time.

Carrying out scientific classification of casualties and accurately assessing the severity of injuries are key issues in post-disaster emergency response. Currently, some scholars have conducted in-depth discussions on this issue [31–34]. For instance, Na and Banerjee [32] classified casualties based on on-site diagnosis and considered the severity of casualties, vehicle capacity, and the available resources at each shelter, aiming to minimize transportation costs. Mills et al. [33] conducted a study that incorporated survival rates, service times, medical resource effectiveness, and injury deterioration for different types of casualties, to maximize the expected number of survivors. Liu et al. [34] considered casualty classification, injury deterioration, and medical resource constraints, and proposed a bi-objective optimization model to determine the optimal locations of temporary medical services and corresponding service allocation plans. However, the above studies have not fully considered the influence of the psychological conditions of casualties on the overall rescue system. In practice, earthquakes often cause casualties to experience psychological trauma during the rescue process, which may disrupt the orderly progress of rescue operations. Therefore, this study incorporates the psychological cost of casualties into the model to provide a more comprehensive reflection of the actual needs in post-disaster rescue efforts.

### 2.3 Psychological factors on disaster relief logistics

Earthquake disasters not only cause physical harm to victims but also result in severe psychological trauma, which can significantly affect the success of rescue operations. Therefore, considering the psychological factors of casualties in emergency response is necessary and practical. In recent years, the psychological state of casualties has attracted the attention of some scholars in emergency rescue [9,35–41]. For example, Hu et al. [35] developed a mixed-integer linear program to address the issue of temporary placement of injured evacuees, aiming to minimize psychological penalty costs, psychological intervention costs, and expenses related to transportation and housing construction. Saffer et al. [39] introduced the psychological cost function into the disaster victim transportation and resource allocation model. Yu et al. [40] represented the psychological suffering of disaster victims through cost losses to enhance the efficiency and fairness of emergency material distribution, and developed a multi-period allocation model for emergency supplies that accounts for such suffering. Yang et al. [41] developed a two-stage satisfaction-oriented optimization model for emergency logistics under earthquake disasters, integrating survivors’ psychological risk perception and urgency of needs to improve resource allocation and disaster response efficiency. The aforementioned studies generally treat emergency medical rescue problems as deterministic and do not adequately account for the pervasive uncertainty present in emergency environments. In reality, disruptions in post-disaster information flow make it difficult to obtain timely, accurate data from affected areas, resulting in unavoidable uncertainty in casualty numbers. This study explicitly incorporates such uncertainty into the modeling framework.

### 2.4 Disaster relief problem based on robust optimization

In post-disaster emergency logistics operations, various sources of uncertainty are pervasive, prompting researchers to widely adopt robust optimization to address these challenges. Currently, robust optimization approaches used in disaster response planning can be broadly categorized into two main types: scenario-based robust optimization and interval-based robust optimization.

In scenario-based robust optimization, researchers typically construct a set of possible disaster scenarios and search for the optimal solution under the worst-case scenario, thereby enhancing the reliability of decisions across different disaster conditions. For example, Sun et al. [42] proposed a scenario-based robust bi-objective optimization model that comprehensively considers the location of medical facilities, transportation of casualties, and distribution of relief supplies with diversion under uncertainty in the number of casualties. Alizadeh et al. [43] examined the location of medical facilities and the allocation of casualties under uncertainty in casualty numbers and transportation capacity, and proposed a scenario-based two-stage robust stochastic optimization model. Amani et al. [44] proposed a data-driven, hybrid scenario-based robust method to solve the mixed-integer second-order cone programming model under uncertain conditions, such as facility disruption probability, number of injured people, transportation time, and rescue needs. Yang et al. [45] considered the risks of facility disruption, scheduling multiple blood products, and evacuating multiple types of casualties, and they adopted a scenario-based robust approach to mitigate the disruption risk of temporary medical centers under various disruption scenarios. Yin and Xu [46] proposed a robust two-stage optimization model to address the site selection and evacuation planning problems in disaster scenarios under uncertain conditions, including rescue facility capacity, demand, and transportation connection availability.

In contrast, interval-based robust optimization characterizes parameter uncertainty using interval uncertainty sets and constructs robust models on this basis. Zhang and Jiang [47] developed a robust location optimization model for emergency medical service stations under uncertain conditions, including the number of emergency calls and the maximum number of concurrent calls. Ahmadi et al. [48] developed a two-stage robust model to cope with disasters, taking into account the uncertainty of demand and travel time. Xu et al. [49] proposed a bi-objective model for locating emergency logistics facilities under uncertain conditions, including cost, time, demand, and road conditions, and also constructed robust models for two uncertain environments. Zhang et al. [50] constructed a robust optimization model integrating medical facility site selection, resource allocation, and casualty transportation under uncertainty regarding the number of casualties and transportation time. In summary, although scenario-based robust optimization can capture various disaster situations, it requires constructing a large number of scenarios, which leads to a high computational burden. It may still not fully cover all possible worst-case conditions. In contrast, interval-based robust optimization only requires the parameter bounds to form the uncertainty set, which aligns better with the limited data availability in post-disaster environments and offers higher computational efficiency. Therefore, this study adopts an interval-based robust approach to model the uncertainty in casualty numbers and develops a bi-objective optimization model for emergency facility location and casualty transportation.

Table 1 summarizes key related works and their main features to highlight the differences between this study and existing research.

**Table 1:** Comparison of relevant literature with our research.

Article	Facility location	Casualty transportation	Consider injury	Psychological factors	Uncertainty type	Uncertainty approach	Objective function
Gu et al. [18]	√	√	√				The number of patients
Liu et al. [16]	√					Distributionally robust	Total cost
Murali et al. [17]	√				Demand	Stochastic programming	The number of people treated
Jin et al. [51]	√	√	√				The number of patients
Hu et al. [35]	√	√		√			Total cost
Zhang et al. [50]	√	√	√		Casualty number transportation time	Robust optimization	Total cost
Yin and Xu [46]	√	√			Supply demand	Two-stage recoverable robust optimization	Total cost
Sun et al. [42]	√	√	√		Casualty number	Scenario-based robust optimization	Deprivation cost Operation cost
Alizadeh et al. [43]	√	√	√		Casualty number transportation capacity	Two-stage robust stochastic optimization	Travel cost
Amani et al. [44]	√	√	√		Casualty number scenario-based time demand	Data driven hybrid scenario-based robust	Total cost
This paper	√	√	√	√	Casualty number	Robust optimization	ISS Psychological cost

The gaps between this study and existing research are as follows:

- Most studies examined facility location, casualty transport, and classification as separate issues. Although some considered these factors together, few took into account the dynamic changes in casualty deterioration over time. This paper incorporates these factors into the model to accurately reflect the real-time changes in the casualty’s injury condition.
- Current research mainly focuses on minimizing transportation time and cost or maximizing the number of surviving casualties as the main optimization objectives. However, it pays less attention to the psychological state of the casualties, particularly the relationship between their psychological state and other objective factors. This paper constructs a bi-objective optimization model to minimize the total ISS and psychological costs of the casualties.

## 3 Problem definition and formulation

**Fig 1:**
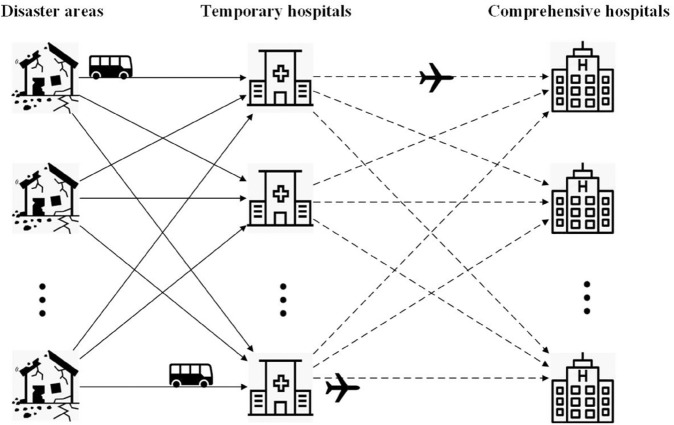
The illustration of the emergency rescue network.

We construct a three-level rescue chain comprising disaster areas, temporary hospitals, and comprehensive hospitals. After an earthquake, a large number of casualties often appear in the disaster area. The first responders who arrive at disaster areas classify the casualties into three categories: mild, moderate, and serious, according to the ISS. To improve rescue efficiency, nearby health centers or clinics are selected as candidates for temporary hospitals, and comprehensive hospitals located in safer, more distant areas serve as candidates for advanced treatment. Rescue vehicles transport all casualties from the disaster area to temporary hospitals for initial treatment. All the casualties are then transferred by helicopter to comprehensive hospitals for further medical care. The structure of the rescue network for casualty transportation is shown in Fig 1.

According to [5], casualties can be classified into three categories: serious casualties (ISS≥50), moderate casualties (20≤ISS<50), and mild casualties (*ISS*<20). Studies indicate that when the injury severity score is 50 or higher (serious casualties), approximately 75% of deaths occur within the first hour after injury. For casualties with scores between 20 and 50 (moderate casualties), most deaths occur within 1 to 6 hours after injury. Whereas for those with scores below 20 (mild casualties), more than half of the deaths occur between 6 hours and one week after injury. Based on this, [6] used a linear function to describe the evolution of ISS over time for casualties with different injury severities. They assumed that the deterioration rates for serious and mild casualties were fixed constants. However, in reality, the deterioration rate does not remain constant. [52] and [28] also pointed out that the deterioration rate changes over time. In the first few hours after injury, the casualties may face acute life-threatening conditions such as blood loss, infection, internal organ damage, and shock. At this stage, their bodies are unable to effectively cope with the trauma, and their condition typically deteriorates rapidly. As time passes, although the injury may continue to worsen, the initial acute responses gradually subside, and the rate of deterioration slows significantly. Nevertheless, without timely treatment, the injury can still lead to death. Therefore, the deterioration rate varies over different time periods. In addition, due to factors such as massive blood loss, multiple organ failure, and severe infection, serious casualties face much higher risks than those with moderate or mild casualties. In contrast, the condition of the mild casualties deteriorates more slowly, with a lower risk [6,53,54].

Building on the above medical principles, we construct a time-varying piecewise function *p*(*t*) (see Eq (1)) to capture the nonlinear and stage-specific deterioration of casualties.

$$p(t)=\left\{ \begin{array}{cc} 0.5{\left(t/T\right)}^{2} & t\leq T\\ 1-0.5e^{2\left(1-t/T\right)} & t>T \end{array}\right.$$ (1)

The first segment uses a quadratic form $0.5(t/T{)}^{2}$, which describes the early pattern in which a patient’s condition worsens slowly at first and then accelerates. The second segment adopts an exponential form $1-0.5e^{2\left(1-t/T\right)}$, representing the later stage in which the rate of deterioration gradually decreases and eventually stabilizes. To avoid unrealistic jumps in the deterioration trajectory and to ensure smoothness in the optimization model, we impose continuity and differentiability at the connection point *t* = *T*, namely:


$$\lim_{t\to T^{-}}p(t)=\lim_{t\to T^{+}}p(t)=0.5,\;p_{-}^{\prime }(T)=p_{+}^{\prime }(T)=\frac{1}{T},$$

This construction is consistent with clinical observations that deterioration tends to be faster in the early stage and slows down afterward. It also enhances both the realism and mathematical tractability of the model. Overall, the proposed function is well aligned with the medical evidence reported in [5,6,28,52], and provides a more accurate representation of the complex, time-varying deterioration of casualties in disaster medical settings.

**Fig 2:**
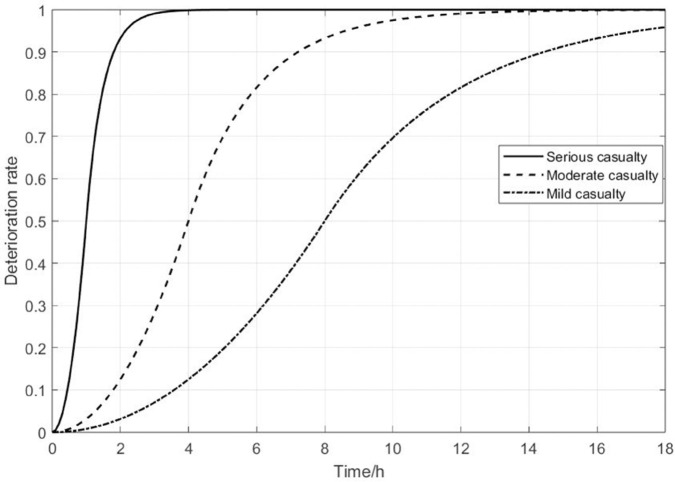
The change in casualty deterioration rate over time.

Moreover, the variable *T* in Eq 1 represents the expected death time of casualties, and also reflects the severity of casualties; a smaller value of *T* corresponds to a more severe injury. To distinguish between the pre-treatment and post-treatment conditions of different types of casualties, let $T=\{T_{s}^{\prime },T_{s}^{\prime \prime }\}$, where $T_{s}^{\prime }$ and $T_{s}^{\prime \prime }$ represent the expected time to death before and after treatment for type *s*, respectively. Here, the subscript *s* indexes the casualty type, with S={s|s=a,b,c} representing serious (*a*), moderate (*b*), and mild (*c*) casualties. It is worth noting that medical intervention can slow down the deterioration of a casualty’s condition and increase the probability of survival; therefore, $T_{s}^{\prime \prime }>T_{s}^{\prime }$. Fig 2 illustrates the change trend of the ISS of casualties over time of different casualty types. The figure shows that the deterioration rate of serious casualties is always higher than that of moderate casualties and mild casualties.

The model proposed in this paper is based on the following assumptions: (1) Each temporary hospital can serve multiple disaster areas simultaneously, and each disaster area can be assigned to multiple temporary hospitals. (2) Rescue vehicles and rescue helicopters are homogeneous resources, and their fixed capacities are considered. The speed of the rescue vehicle remains constant during the journey. The total number of available helicopters and vehicles is limited. (3) The time required to quickly treat the casualties in temporary hospitals is not considered. (4) The locations and numbers of disaster areas, candidate temporary hospitals, and candidate comprehensive hospitals are known. (5) Referring to [52], the deterioration rate of casualties will be significantly reduced after receiving treatment in the temporary hospital.

### 3.1 Notation descriptions

Notations and definitions used throughout the paper are listed in Table 2.

**Table 2:** Notation descriptions.

Notation	Descriptions
**Sets**	
*I*	Set of disaster areas, i∈I
*J*	Set of candidate temporary hospitals, j∈J
*K*	Set of candidate comprehensive hospitals, k∈K
*S*	Set of casualty types, S={s|s=a,b,c}, *a* for serious casualty, *b* for moderate casualty, *c* for mild casualty
**Parameters**	
$\gamma_{i}$	The number of available vehicles in disaster areas *i*
$\lambda_{j}$	The number of available helicopters in temporary hospitals *j*
*e^g^*	The carrying capacity of each emergency rescue vehicle
*e^h^*	The carrying capacity of each helicopter
$\omega_{s}$	Priority given to serious casualties, moderate casualties and mild casualties, respectively
*q*_*is*_	Number of casualties type *s* in the disaster area *i*
*t*_*ij*_	Travel time between disaster area *i* and temporary hospital *j* by vehicle
*t*_*jk*_	Travel time between temporary hospital *j* and comprehensive hospital *k* by helicopter
*C*	Capacity of temporary hospital for casualties
*Q*	Capacity of comprehensive hospital for casualties
*p*_*ijs*_	The deterioration rate of casualties type *s* transported from disaster area *i* to candidate temporary hospital *j*
*u*_*jks*_	The deterioration rate of casualties type *s* transported from candidate temporary hospital *j* to candidate comprehensive hospital *k*
$\alpha_{s}$	The psychological burden coefficient of casualties type *s* during the transportation from the disaster area to the temporary hospital
$\beta_{s}$	The psychological burden coefficient of casualties type *s* during transportation from temporary hospitals to comprehensive hospitals
$T_{s}^{\prime }$	The expected death time of casualties type *s* without treatment
$T_{s}^{\prime \prime }$	The expected death time of casualties type *s* after treatment
$\Gamma_{s}$	Budget of the uncertainty
**Decision Variables**	
*U*_*j*_	1 if temporary hospital *j* is selected, 0 otherwise
$V_{k}$	1 if comprehensive hospital *k* is selected, 0 otherwise
*x*_*ijs*_	Number of casualties type *s* transported from disaster area *i* to temporary hospital *j*
*y*_*jks*_	Number of casualties type *s* transported from temporary hospital *j* to comprehensive hospital *k*

### 3.2 Psychological costs of panic of casualties

Earthquake disasters not only cause serious physical injuries to casualties but also have negative effects on their psychology. For example, anxiety and despair caused by delays in rescue or treatment, as well as fear of recurring disaster, may increase over time, making it more difficult to carry out rescue efforts. Existing studies have tried to describe the psychological condition of casualties using psychological cost functions. [9] divided the psychological cost into multiple stages, including the accident site, shelter, medical institutions, and psychological burden during transportation. However, they did not consider the impact of the treatment of casualties on the psychological state. Based on interviews with medical personnel and casualties from the Wenchuan and Yushu earthquakes, [55] found that as the casualties receive treatment, their psychological burden gradually decreases. Moreover, under the same conditions, the psychological costs of the same type of casualties are similar. Based on the above facts, we define a psychological cost function for different types of casualties. The defined psychological costs include: (1) the psychological costs of transporting all types of casualties from disaster areas to temporary hospitals; (2) the psychological costs of transporting casualties from temporary hospitals to comprehensive hospitals. The calculation of psychological costs in this study is as follows:

The psychological cost of transporting casualties from the disaster area to temporary hospitals is

$$R_{1}=\alpha_{s}t_{ij}x_{ijs}$$ (2)

The psychological cost of transporting casualties from the temporary hospitals to comprehensive hospitals is

$$R_{2}=\beta_{s}t_{jk}y_{jks}$$ (3)

Both $\alpha_{s}$ and $\beta_{s}$ in Eqs 2 and 3 represent the psychological burden coefficients of casualties. A larger value indicates a heavier psychological burden. As casualties receive treatment, their psychological burden gradually decreases. Therefore, the burden is greater during the transfer from the disaster site to the temporary hospital than during the transfer from the temporary hospital to the comprehensive hospital, that is, $\alpha_{s}>\beta_{s}$. In addition, [55] shows that the more severe the injury, the heavier the psychological burden. Hence, for different types of casualties, we have $\alpha_{a}>\alpha_{b}>\alpha_{c}$ and $\beta_{a}>\beta_{b}>\beta_{c}$.

### 3.3 Mathematical formulation

$$minf_{1}=\sum_{i}\sum_{j}\sum_{s}\omega_{s}p_{ijs}x_{ijs}+\sum_{j}\sum_{k}\omega_{s}u_{jks}y_{jks}$$ (4)

$$minf_{2}=\sum_{i}\sum_{j}\sum_{s}R_{1}+\sum_{j}\sum_{k}\sum_{s}R_{2}$$ (5)

$$p_{ijs}=\left\{ \begin{array}{cc} 0.5{\left(t_{ij}/T_{s}^{\prime }\right)}^{2} & t_{ij}\leq T_{s}^{\prime }\\ 1-0.5e^{2\left(1-t_{ij}/T_{s}^{\prime }\right)} & t_{ij}>T_{s}^{\prime } \end{array}\right.$$ (6)

$$u_{jks}=\left\{ \begin{array}{cc} 0.5{\left(t_{jk}/T_{s}^{\prime \prime }\right)}^{2} & t_{jk}\leq T_{s}^{\prime \prime }\\ 1-0.5e^{2\left(1-t_{jk}/T_{s}^{\prime \prime }\right)} & t_{jk}>T_{s}^{\prime \prime } \end{array}\right.$$ (7)

$$\sum_{i}\sum_{s}x_{ijs}\leq U_{j}C$$ (8)

$$\sum_{j}\sum_{s}y_{jks}\leq V_{k}Q$$ (9)

$$\sum_{j}\sum_{s}x_{ijs}\leq \gamma_{i}e^{g}$$ (10)

$$\sum_{k}\sum_{s}y_{jks}\leq \lambda_{j}e^{h}$$ (11)

$$U_{j}\leq \sum_{i}\sum_{s}x_{ijs}$$ (12)

$$V_{k}\leq \sum_{j}\sum_{s}y_{jks}$$ (13)

$$\sum_{j}x_{ijs}=q_{is}$$ (14)

$$\sum_{i}x_{ijs}=\sum_{k}y_{jks}$$ (15)

$$U_{j},V_{k},\in \{0,1\},\forall j\in J,\forall k\in K$$ (16)

$$x_{ijs},y_{jks}\geq 0,\text{and interger}$$ (17)

Eq (4) is the objective function to minimize the total weighted ISS of casualties. Eq (5) is the objective function to minimize the sum of the psychological cost for casualties. It consists of two components: the psychological cost incurred during the transfer from the disaster area to temporary hospitals (Eq 2), and the psychological cost incurred during the transfer from temporary hospitals to comprehensive hospitals (Eq 3). Eq (6) describes the ISS deterioration of casualty type *s* during the first transfer stage (from the disaster area to temporary hospitals), based on the pre-treatment expected death time $T_{s}^{\prime }$. Eq (7) characterizes the ISS deterioration during the second transfer stage (from temporary hospitals to comprehensive hospitals), using the post-treatment expected death time $T_{s}^{\prime \prime }$. Constraint (8) ensures that the number of casualties transported from the disaster area to the temporary hospital does not exceed its maximum capacity. Constraint (9) ensures that the number of casualties transported from the temporary hospital to the comprehensive hospital does not exceed its maximum capacity. Constraints (10) and (11) guarantee that the number of casualties transported from the disaster area is no greater than the helicopter capacity and the number of casualties transported from the temporary hospital is no greater than the vehicle capacity. Constraints (12) and (13) ensure that at least one casualty arrives at the selected temporary hospital and comprehensive hospital. Constraint (14) guarantees that all casualties in the disaster area are transported to temporary hospitals. Constraint (15) ensures that all serious casualties in temporary hospitals are transferred to comprehensive hospitals. Finally, all decision variables are defined in constraints (16) and (17).

## 4 Solution approach

### 4.1 Robust model formulation using robust optimization method

Since earthquakes occur suddenly and are destructive, it is difficult to accurately estimate the number of casualties in the disaster area. To solve this problem, we employ the robust optimization method proposed by [11] to address the uncertainty in the number of casualties. According to their method, we define the uncertainty in casualty numbers, *q*_*is*_, as an independent, bounded interval with a range of $\left[{\bar{q}}_{is}-{\hat{q}}_{is},{\bar{q}}_{is}+{\hat{q}}_{is}\right]$. Where notation ${\bar{q}}_{is}$ represents the nominal, while ${\hat{q}}_{is}$ represents the maximum deviation from ${\bar{q}}_{is}$. Then, we introduce an uncertainty budget $\Gamma_{s}\in [0,1]$ to control the actual deviation. Applying parameter $\Gamma_{s}$ adjusts the degree of protection against uncertainty, the robust counterpart of constraint (14) can be rewritten in the following form:

$$\sum_{j}x_{ijs}\geq {\bar{q}}_{is}+\max_{\eta \in Z}\left(\sum_{s}{\hat{q}}_{is}\eta_{is}\right)$$ (18)

Here, $\eta_{is}=\frac{q_{is}-{\bar{q}}_{is}}{{\hat{q}}_{is}}$ is the scaled deviation and $Z=\{\eta ||\eta |\leq 1,\sum_{i}\eta_{is}\leq \Gamma_{s}\}$. We substitute the dual problem of the protection function $\max_{\eta \in Z}\left(\sum_{s}{\hat{q}}_{is}\eta_{is}\right)$ into constraint (18). Thus, when uncertainty affects only the right-hand side value, the robust counterpart of constraint (14) can be rewritten in the following form:

$$\sum_{j}x_{ijs}\geq {\bar{q}}_{is}+{\hat{q}}_{is}\Gamma_{s}$$ (19)

Where $\Gamma_{s}=0$ represents the nominal case and $\Gamma_{s}=1$ represents the worst case. Increasing $\Gamma_{s}$ will result in a more conservative solution because the model contains a greater degree of uncertainty. The decision-maker can set an appropriate value for $\Gamma_{s}$ based on his attitude to risk.

Therefore, the robust counterpart of our proposed model is depicted as follows:


$$minf_{1}=\sum_{i}\sum_{j}\sum_{s}\omega_{s}p_{ijs}x_{ijs}+\sum_{j}\sum_{k}\omega_{s}u_{jks}y_{jks}$$

$$minf_{2}=\sum_{i}\sum_{j}\sum_{s}R_{1}+\sum_{j}\sum_{k}\sum_{s}R_{2}$$

s.t. (2)–(3), (6)–(13), (15)–(17), (19)

### 4.2 Bi-objective model transformation using the ε-constraint method

Due to the conflict between the two objective functions in the proposed model, it is impossible to obtain a solution that optimizes both objectives simultaneously. Therefore, in order to obtain non-dominant solutions or Pareto solution sets, a multi-objective solution process must be adopted.

Common multi-objective optimization methods include the weighted sum method, goal programming, and the ε−constraint method [56]. However, [57] found that the weighted sum method often fails to generate effective solutions, as the results are highly sensitive to the assigned weights. In addition, goal programming involves a relatively complex modeling process and introduces a certain degree of subjectivity. In contrast, the ε−constraint method does not require additional variables or weight, nor does it need to account for differences in units or magnitudes among objectives. Instead, it generates an efficient solution set by simply controlling the number of grid points within the objective function range, making it both convenient and computationally efficient [58]. Therefore, this paper adopts the ε−constraint method to transform the bi-objective optimization problem into a single-objective optimization problem. The transformation process is as follows:

(1)Determine the optimal values of each objective function, $f_{1}^{\;*}$ and $f_{2}^{\;*}$, under the given constraints.

(2)Take *f*_1_ as the main objective function and convert *f*_2_ into a constraint, requiring that *f*_2_ remain within an acceptable threshold $\varepsilon \geq f_{2}^{\;*}$. By adjusting the value of ε, a set of Pareto optimal solutions can be obtained. The transformed model is shown as follows:


$$minf_{1}$$

$$f_{2}\leq \varepsilon$$

s.t. (2)–(3), (6)–(13), (15)–(17), (19)

## 5 Model application and computational experiments

To evaluate the performance of the proposed model, we conduct a case study based on the emergency logistics response to the 2010 Yushu earthquake in Qinghai. The model is implemented and solved using IBM ILOG CPLEX 12.10 on a PC equipped with a 2.00 GHz AMD R7 processor and 16 GB of RAM.

### 5.1 Parameter setting

This study is based on the Yushu County earthquake in Qinghai Province. The relevant parameter settings are derived from actual data and supplemented with simulated data [6,53,59,60]. A total of 8 towns affected by the earthquake are selected as disaster areas, marked as I1 to I8. Table 3 presents the number of casualties in each disaster area [53]. In addition, 6 health centers close to the disaster areas are chosen as candidate temporary hospitals, denoted J1 to J6, and 4 large hospitals are chosen as candidate comprehensive hospitals, marked as K1 to K4. The capacity of each candidate’s temporary hospital is 850 units, and the capacity of each candidate’s comprehensive hospital is 1200 units [53]. In each disaster area, 100 Dongfeng EQ240 rescue vehicles are deployed, each capable of transporting 6 casualties. Additionally, each temporary hospital is equipped with 60 MI-17 helicopters, each capable of accommodating up to 12 casualties [34,61]. Tables 4 and 5 present the transportation time of vehicles from each disaster area to temporary hospitals and the flight time of helicopters from temporary hospitals to comprehensive hospitals, respectively [53]. To describe the psychological state of different types of casualties and their changes before and after treatment, we set the psychological burden coefficients ($\alpha_{s}$ and $\beta_{s}$) to 6, 2.5, 1.5, and 3.5, 1.5, 0.5, respectively. Some of these parameter values are drawn from relevant settings in the existing literature [9,55], while the others are reasonably assumed based on real-world post-disaster emergency scenarios. These settings are also consistent with clinical observations in the field of psychological trauma, which ensures their rationality and applicability. According to [62] and [55], the $T_{s}^{\prime }$ values before receiving treatment at temporary hospitals are set to 1 hour for serious casualties, 4 hours for moderate casualties, and 12 hours for mild casualties. After receiving treatment, the corresponding $T_{s}^{\prime \prime }$ values are set to 2, 8, and 24 hours, respectively. Moreover, the weights of serious, moderate, and mild casualties are set to 3, 2, and 1, respectively.

**Table 3:** Nominal number of casualties in disaster areas.

Disaster areas	I1	I2	I3	I4	I5	I6	I7	I8
Serious casualty	137	52	78	111	122	54	46	40
Moderate casualty	77	134	115	88	106	72	120	66
Mild casualty	214	235	228	223	202	287	298	329

**Table 4:** Transportation time between disaster areas and candidate temporary hospitals (unit: h).

Disaster areas	Temporary hospitals					
	J1	J2	J3	J4	J5	J6
I1	2.57	3.17	3.14	4.13	4.36	4.53
I2	2.51	3.11	3.09	3.98	4.28	4.55
I3	2.71	3.31	3.28	3.27	4.27	4.45
I4	3.08	3.00	3.77	3.12	4.03	4.42
I5	2.42	3.18	3.27	3.38	4.60	4.78
I6	2.83	3.30	3.15	3.22	4.20	4.50
I7	2.33	3.55	3.37	3.67	4.55	4.86
I8	2.78	3.15	3.00	3.50	4.40	4.75

**Table 5:** Transportation time between candidate temporary hospitals and candidate comprehensive hospitals (unit: h).

Temporary hospitals	Comprehensive hospitals			
	K1	K2	K3	K4
J1	0.55	0.98	0.60	0.78
J2	0.59	2.34	0.76	0.80
J3	0.70	2.40	0.82	0.85
J4	0.61	2.36	0.78	0.81
J5	1.00	1.26	1.05	1.24
J6	0.83	2.18	0.98	1.17

### 5.2 Calculation results of the deterministic problem

**Fig 3:**
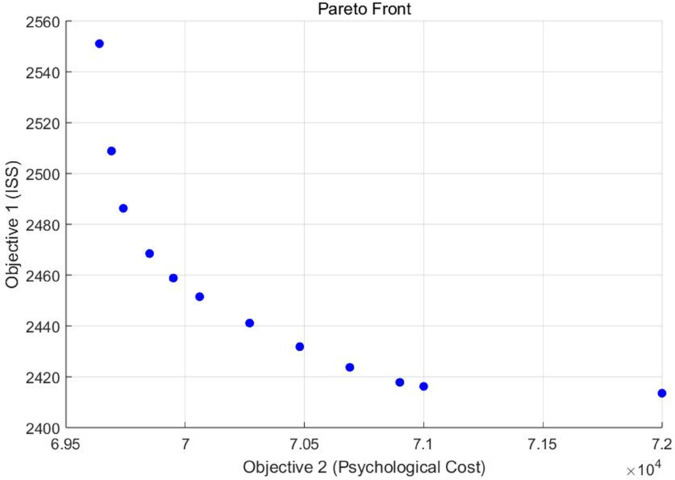
Trade-off between minimizing ISS and psychological cost.

Using the nominal value, the minimum values of the two objective functions are $f_{1}^{\;*}=2413.5$ and $f_{2}^{\;*}=69637$, respectively. Next, *f*_1_ is treated as the main objective function, and the psychological cost of casualties is converted into a constraint by setting $\varepsilon \geq f_{2}^{\;*}$, resulting in a set of Pareto optimal solutions. Table 6 presents the objective function values of the deterministic bi-objective model for different values of ε. The deviation shown in the fourth column is calculated as $(f_{1}\;-\;f_{1}^{\;*})/f_{1}^{\;*}$. To provide a more intuitive understanding, the trade-off between the two objectives is illustrated in Fig 3 by appropriately selecting values of ε. It can be observed that as the psychological cost increases, the total ISS of casualties decreases. However, once the psychological cost exceeds a certain threshold, further increases have little impact on the total ISS. This phenomenon also illustrates the need to make a trade-off between the ISS and psychological costs.

**Table 6:** The results with different values of ε.

NO.	ε(Psychological cost)	$f_{1}$(ISS)	Deviation (%)
1	69630	–	–
2	69640	2551.10	5.70
3	69850	2468.50	2.28
4	70060	2451.50	1.57
5	70270	2441.10	1.14
6	70480	2431.80	0.76
7	70690	2423.70	0.42
8	70900	2417.80	0.18
9	71000	2416.20	0.01
10	72000	2413.50	0.00

As shown in Table 6, when 69640≤ε≤75000, the objective function value *f*_1_ decreases as ε increases. However, when ε≥75000, the objective function value *f*_1_ remains unchanged. When the ε value is too small, such as ε=69630, the model yields no feasible solution. Therefore, a conflict exists between the objective functions *f*_1_ and *f*_2_. It is impossible for decision-makers to find the best solution for both objectives simultaneously. In real-world humanitarian rescue operations, they must balance physiological treatment and psychological costs according to the priorities at different rescue stages. For example, in the early emergency phase (such as the 72-hour “golden rescue period” after an earthquake), decision-makers should choose a larger ε to prioritize physiological treatment outcomes. In contrast, during the mid-to-late stages of rescue (such as the transitional assistance stage), when physiological conditions are relatively stable while psychological trauma becomes more prominent, selecting a smaller ε is more appropriate.

**Fig 4:**
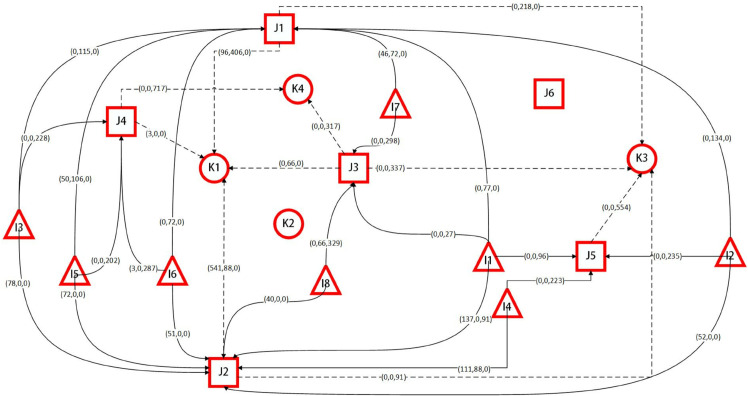
Decision scheme of the deterministic model based on nominal data.

Fig 4 presents the casualty transportation scheme derived from the deterministic model. In the figure, triangles represent disaster sites, squares represent temporary hospitals, and circles represent comprehensive hospitals. Solid lines denote the routes from disaster sites to temporary hospitals, while dashed lines indicate the routes from temporary hospitals to comprehensive hospitals. As shown in the figure, the final location-allocation result includes five temporary hospitals and three comprehensive hospitals. The three values in parentheses indicate the numbers of serious casualties, moderately casualties, and mild casualties transferred along each route. For example, from disaster site I1 to temporary hospital J2, the numbers of serious, moderate, and mild casualties are 137, 0, and 91, respectively; while from temporary hospital J1 to comprehensive hospital K1, the corresponding numbers are 96, 406, and 0, respectively.

### 5.3 Calculation results of the uncertainty problem

To investigate the impact of uncertain parameters on the main objective function, we perform some numerical experiments based on the robust model.

Different values of $\Gamma_{s}$ are used in this study to represent the degree of information uncertainty in the post-disaster rescue process. Specifically, $\Gamma_{s}=0$ represents a completely deterministic scenario, where all parameters (such as the number of casualties) are known. In this case, the robust model is equivalent to a deterministic model. At the other extreme, $\Gamma_{s}=1$ captures a highly uncertain environment, such as the immediate aftermath of a major earthquake when communications are disrupted, roads are severely damaged, and timely casualty information is unavailable. In such cases, decision makers usually need to adopt more conservative strategies to deal with the worst-case scenario. Intermediate values ($\Gamma_{s}\in (0,1)$) correspond to moderate uncertainty. For example, during the mid-to-late rescue stage, with the aid of drone reconnaissance, field feedback, or partial restoration of communications, casualty estimates become more reliable. In this case, choosing a moderate $\Gamma_{s}$ can achieve a trade-off between robustness and the solution’s conservatism. A risk-seeking decision maker tends to choose a smaller level of uncertainty $\Gamma_{s}$, but which, in turn, exposes them to the potential losses arising from such uncertainty. In contrast, a risk-averse decision maker prefers a larger uncertainty level $\Gamma_{s}$ to secure a higher probability of obtaining an effective and feasible rescue plan. A risk-neutral decision maker, meanwhile, typically adopts a moderate or balanced level of uncertainty $\Gamma_{s}$. Some studies in emergency management have also adopted similar $\Gamma_{s}$ value ranges [50,53].

In the sensitivity analysis, we vary the uncertainty budget to assess how uncertainty in casualty numbers influences the objective function value. For each given uncertainty budget, the data variability was set to 5%, 15%, and 20%.

**Fig 5:**
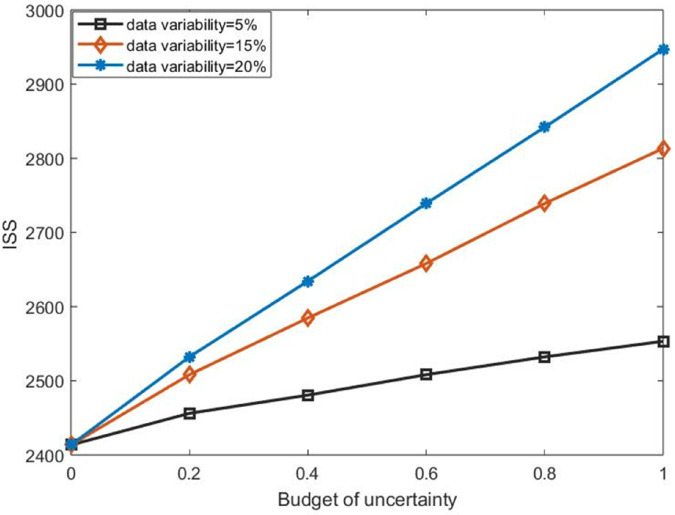
Changes in ISS with respect to uncertainty budget under different data variability.

Fig 5 describes the impact of changes in the uncertainty parameters of the number of casualties on the value of the main objective function under different data variability and uncertainty budgets. As shown in Fig 5, the results indicate that ISS increases as the uncertainty budget or data variability increases and reaches the worst value when these parameters are at their maximum. This shows that the higher the uncertainty in the number of casualties, the larger the total ISS, and the lower the data variability, the more robust the model.

Table 7 shows the impact of the psychological factors of casualties on the rescue effect under casualty number uncertainty. The results indicate that, as ε increases, the objective function ISS decreases, and when ε reaches a certain level, ISS no longer changes. This conclusion also provides important insights for real-world management. During the rescue process, managers should pay appropriate attention to psychological costs, as this can enable casualties to receive timely and sufficient psychological support, making them more willing to accept treatment and thereby effectively reducing the overall ISS. However, if excessive attention is devoted to psychological factors, the improvement in the ISS will be limited, and scarce rescue resources may be disproportionately allocated to psychological interventions, crowding out critical medical treatment and ultimately reducing overall rescue efficiency.

**Table 7:** The results in different settings.

Budget of uncertainty $\Gamma_{s}$	Data variability (%)	ε	Main objective value ISS
0.2	5%	71000	2571.90
		72000	2467.60
	20%	74000	2595.00
		75000	2539.20
0.4	5%	72000	2552.40
		73000	2489.60
	20%	78000	2692.20
		79000	2640.60
0.6	5%	73000	2577.20
		74000	2516.80
	20%	82000	2797.80
		83000	2745.20
0.8	5%	74000	2595.00
		75000	2539.20
	20%	86000	2902.50
		87000	2848.00
1	5%	75000	2606.10
		76000	2558.00
	20%	90000	3007.00
		91000	2951.80

Table 8 presents the impact of different weight settings on the main objective function value and the optimal location scheme. As shown, the objective function value increases with casualty weights, with the weight of serious casualties exerting the most significant influence. In practical rescue operations, this implies that serious casualties should be prioritized in transportation and medical resource allocation, as they deteriorate more rapidly and have lower tolerance for delayed treatment. This finding aligns with established triage principles in emergency medical response, such as the START (Simple Triage and Rapid Treatment) method and the “red, yellow, green, and black” four-level classification widely used in China’s earthquake rescue, both of which emphasize that limited resources should first be allocated to serious casualties. The proposed model not only provides quantitative validation for these triage principles but also offers policy-relevant insights for emergency management authorities, enabling them to allocate rescue forces and set priority sequences more scientifically, thereby enhancing the efficiency and effectiveness of emergency medical response.

**Table 8:** Impact of the weight on main objective function value.

Priority			Budget of uncertainty	Data variability (%)	ISS of serious casualties	ISS of moderate casualties	ISS of mild casualties	Main objective value ISS	Location decisions	
$\omega_{3}$	$\omega_{2}$	$\omega_{1}$							Temporary hospitals	Comprehensive hospitals
1	1	1	0	5-20	662.96	169.10	87.34	919.40	J1,J2,J3,J4,J5	K1,K3,K4
			0.3	5	662.96	169.10	87.34	919.40	J1,J2,J3,J4,J5	K1,K3,K4
				15	698.55	177.80	95.36	971.71	J1,J2,J3,J4,J5,J6	K1,K2,K3,K4
				20	708.82	180.84	98.40	988.06	J1,J2,J3,J4,J5,J6	K1,K2,K3,K4
			0.7	5	690.08	176.14	93.72	959.94	J1,J2,J3,J4,J5	K1,K3,K4
				15	737.88	190.02	107.24	1035.10	J1,J2,J3,J4,J5,J6	K1,K2,K3,K4
				20	761.75	196.72	114.09	1072.60	J1,J2,J3,J4,J5,J6	K1,K2,K3,K4
2	2	1	0	5-20	1325.60	338.20	87.58	1751.40	J1,J2,J3,J4,J5	K1,K3,K4
			0.3	5	1353.80	345.25	90.55	1789.60	J1,J2,J3,J4,J5	K1,K3,K4
				15	1396.50	355.60	95.78	1847.90	J1,J2,J3,J4,J5,J6	K1,K2,K3,K4
				20	1417.30	361.68	98.69	1877.70	J1,J2,J3,J4,J5,J6	K1,K2,K3,K4
			0.7	5	1379.80	352.27	94.01	1826.10	J1,J2,J3,J4,J5	K1,K3,K4
				15	1475.20	380.04	107.63	1962.90	J1,J2,J3,J4,J5,J6	K1,K2,K3,K4
				20	1522.90	393.44	114.52	2030.90	J1,J2,J3,J4,J5,J6	K1,K2,K3,K4
3	2	1	0	5-20	1981.70	343.64	88.18	2413.50	J1,J2,J3,J4,J5	K1,K3,K4
			0.3	5	2023.20	350.77	91.38	2465.40	J1,J2,J3,J4,J5	K1,K3,K4
				15	2087.00	360.87	96.63	2544.50	J1,J2,J3,J4,J5,J6	K1,K2,K3,K4
				20	2118.90	366.21	99.55	2584.70	J1,J2,J3,J4,J5,J6	K1,K2,K3,K4
			0.7	5	2061.50	357.96	94.86	2514.30	J1,J2,J3,J4,J5	K1,K3,K4
				15	2209.50	383.18	107.98	2699.70	J1,J2,J3,J4,J5,J6	K1,K2,K3,K4
				20	2283.70	393.93	114.56	2792.20	J1,J2,J3,J4,J5,J6	K1,K2,K3,K4
4	3	1	0	5-20	2647.90	509.28	88.17	3245.40	J1,J2,J3,J4,J5	K1,K3,K4
			0.3	5	2701.70	521.71	91.36	3314.80	J1,J2,J3,J4,J5	K1,K3,K4
				15	2784.40	539.45	96.62	3420.40	J1,J2,J3,J4,J5,J6	K1,K2,K3,K4
				20	2825.90	548.58	99.51	3474.00	J1,J2,J3,J4,J5,J6	K1,K2,K3,K4
			0.7	5	2751.30	534.09	94.85	3380.20	J1,J2,J3,J4,J5	K1,K3,K4
				15	2946.00	573.28	107.99	3627.30	J1,J2,J3,J4,J5,J6	K1,K2,K3,K4
				20	3044.90	590.89	114.56	3750.30	J1,J2,J3,J4,J5,J6	K1,K2,K3,K4

Meanwhile, Table 8 shows that as the uncertainty budget increases, the ISS values of all casualty types also rise, with serious casualties showing the most pronounced increase, followed by moderate and then mild casualties. This finding further suggests that, under uncertain conditions, serious casualties are the most sensitive to resource allocation and should therefore be prioritized. Moreover, changes in weights do not affect the optimal location scheme, highlighting the robustness of the proposed model’s location decisions. Table 8 highlights the following managerial implication: in resource-constrained emergency rescue environments, managers should prioritize allocating limited resources to the treatment of serious casualties in order to maximize the effectiveness of medical rescue operations.

**Fig 6:**
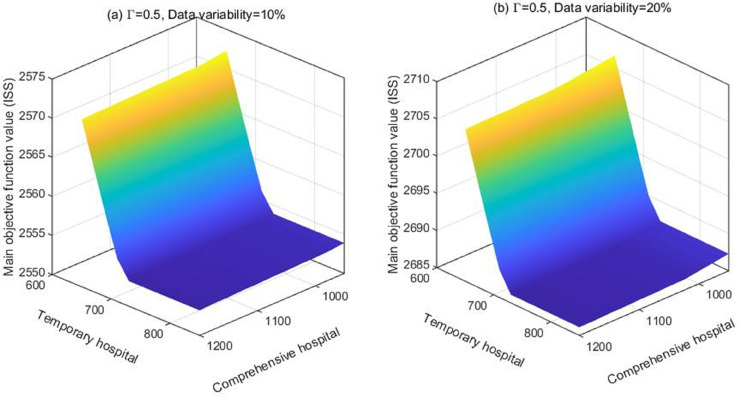
The impact of temporary and comprehensive hospital capacities on the main objective function.

Fig 6 shows the effect of the capacities of temporary hospitals and the comprehensive hospital on the main objective value. We report the results for an uncertainty budget of 0.5, as well as data variability of 10% and 20%. As seen from Fig 6, as the capacity of temporary and comprehensive hospitals increases, the value of the main objective function decreases. Therefore, increasing the capacity of temporary and comprehensive hospitals can improve the efficiency of casualty transfer, thereby effectively reducing the ISS of casualties. Furthermore, comparing the slopes of the curves reveals that the impact of temporary hospital capacity on the main objective function is significantly higher than that of comprehensive hospitals, indicating that temporary hospitals play a more critical role in actual rescue operations. These results provide important implications for management practice. Before a disaster, managers should plan the location and construction scheme of temporary hospitals in earthquake-prone areas, while ensuring adequate medical supplies and well-trained personnel. After a disaster, managers should promptly activate or rapidly establish temporary hospitals according to the disaster situation in order to minimize treatment delays and thereby effectively reduce the casualty rate. Sun et al. [6] suggest that increasing comprehensive hospital capacity improves rescue efficiency, which contrasts with our findings, as their study overlooks the crucial role of temporary hospitals in emergency rescue operations.

To verify the effectiveness of the proposed robust optimization model, we compare it with a traditional deterministic model. Given the data variability and uncertainty budget level, 50 sets of test data are randomly generated, and three test problems of varying sizes are constructed. The performance of the deterministic and robust models is analyzed by comparing the number of feasible solutions and the mean of feasible solutions. Table 9 lists the specific results of each group of experiments. The results show that since the deterministic model cannot meet the constraints under certain uncertain scenarios, it has no solution in multiple test groups. By contrast, the robust model can produce feasible solutions in most cases, further verifying its robustness advantage in dealing with the uncertainty of the number of casualties.

**Table 9:** Comparison of deterministic model and robust model under different problem sizes.

Problem size ∣I∣*∣J∣*∣K∣	Budget of uncertainty	Data variability (%)	Main objective value		Computation time(s)		Average of main objective values		Feasible solutions number	
			D	R	D	R	D	R	D	R
10*8*6	0.3	5	3166.2	3235.8	2.08	2.12	-	3322.7	0	50
$\left(\varepsilon =1.2\times 10^{5}\right)$		15		3341.6		2.13	-	3432.4	0	50
		20		3391.9		2.19	-	3480.0	0	50
	0.7	5		3302.5		2.17	-	3391.6	0	50
		15		3546.0		2.19	-	3639.8	0	50
		20		3665.7		2.20	-	3760.8	0	50
	1	5		3351.6		2.15	-	3444.4	0	50
		15		3695.5		2.22	-	3797.6	0	50
		20		3868.9		2.28	-	3969.9	0	46
12*10*8	0.3	5	3957.9	4041.0	11.86	12.05	-	4352.9	0	50
$\left(\varepsilon =1.5\times 10^{5}\right)$		15		4168.8		14.04	-	4491.9	0	50
		20		4231.0		14.09	-	4558.9	0	50
	0.7	5		4122.4		12.06	-	4445.2	0	50
		15		4422.4		14.09	-	4764.9	0	50
		20		4565.5		14.10	-	4923.6	0	49
	1	5		4182.6		13.87	-	4512.6	0	50
		15		4602.7		14.79	-	4969.3	0	47
		20		4819.0		15.08	-	5191.5	0	44
15*12*10	0.3	5	4674.5	4772.9	36.84	38.21	-	5428.9	0	50
$\left(\varepsilon =1.8\times 10^{5}\right)$		15		4924.8		38.86	-	5603.2	0	50
		20		5000.1		38.93	-	5689.0	0	50
	0.7	5		4872.7		39.19	-	5546.4	0	50
		15		5224.6		39.92	-	5944.3	0	50
		20		5400.1		40.84	-	6141.4	0	50
	1	5		4942.4		39.59	-	5629.6	0	50
		15		5443.3		40.47	-	6197.9	0	50
		20		5701.5		42.86	-	6543.0	0	41

In the core model, we assumed that all casualties transferred to temporary hospitals must eventually be transported to comprehensive hospitals. However, this assumption may be overly restrictive in real-world contexts. Therefore, we extended the assumption to allow mild casualties to be fully treated at temporary hospitals without further transfer to comprehensive hospitals. The results show that under this extended setting, the model achieves a better objective value and requires fewer comprehensive hospital locations. This indicates that reducing secondary transfers of casualties can improve treatment efficiency. At the same time, temporary hospitals help alleviate the burden on comprehensive hospitals by taking on a greater share of treatment tasks. Further sensitivity analyses reveal that the impacts of the casualty number uncertainty parameter on the objective value, the influence of weight changes on the optimal location scheme, and the effect of temporary hospital capacity on the primary objective function remain consistent with the core model. These findings suggest that even with an adjusted transfer strategy, our main conclusions and managerial implications remain robust and broadly applicable.

## 6 Conclusions and future work

Earthquakes can cause a large number of casualties in a short period, making the timely delivery of emergency medical services critically important. This study develops a bi-objective robust optimization model that accounts for uncertainty in casualty numbers, with a focus on medical facility location and casualty transportation within a three-tier rescue chain comprising disaster areas, temporary hospitals, and comprehensive hospitals. The model also accounts for the dynamic deterioration of casualties during transportation. The objective is to minimize the total ISS and the psychological cost of casualties. The robust optimization method proposed by [11] is employed to handle parameter uncertainty using interval data, and the ε−constraint method is used to obtain the exact Pareto front of the bi-objective model. A case study based on the 2010 Yushu earthquake is conducted to validate the effectiveness of the proposed model. The results show that: (1) the greater the uncertainty budget or data variability in casualty numbers, the more significant the impact on total ISS. Decision-makers can adjust the uncertainty parameters to determine satisfactory rescue plans. (2) Compared with mild and moderate casualties, prioritizing the treatment of severe casualties can improve the effectiveness of medical interventions. (3) The capacity of temporary hospitals has a greater impact on ISS than the capacity of comprehensive hospitals. Therefore, the capacity of temporary hospitals should be expanded as much as possible. (4) Paying attention to the casualties’ psychological condition can help improve humanitarian care, but it may come at the expense of rescue efficiency. Therefore, decision makers should make a trade-off between humanitarian care and rescue effectiveness based on their actual preferences.

In addition, in the early chaotic stage after an earthquake, casualty information is often highly uncertain due to communication failures and severe road damage, which corresponds to a high level of uncertainty in the model. Under such conditions, decision-makers should prioritize the treatment of serious casualties in a resource-constrained environment to prevent irreversible deterioration caused by delayed medical care. At the same time, psychological support should not occupy excessive critical resources; otherwise, it may hinder timely treatment and reduce the overall efficiency of the rescue operation. As the rescue operation progresses into a more stable later stage, the level of uncertainty decreases. Under such lower uncertainty, decision-makers can adjust their strategies as needed, providing essential medical treatment while also attending to the psychological and recovery needs of casualties, without significantly impacting the overall effectiveness of the rescue plan.

This study has some limitations. The model only considers the uncertainty of casualties, whereas real-world disasters involve multiple uncertainties, including uncertainty in transportation time, rescue costs, and facility disruptions. Future research can incorporate these factors to better reflect the complexity of actual disaster scenarios. Additionally, the robust optimization method employed in this study relies on limited information, which may result in overly conservative solutions. Distributed robust optimization methods can handle uncertainties more flexibly, striking a balance between conservatism and practicality. Future research can explore this method to improve the accuracy and adaptability of emergency rescue decisions. Finally, this study adopted a classical psychological cost function widely used and validated in the literature. However, the function itself was not substantially advanced in terms of structure or estimation. Future research may explore alternative formulations to capture better the impact of casualties’ psychological states on rescue effectiveness.
